# Chasing Methuselah: adult inducible GHRKO mice

**DOI:** 10.18632/aging.204016

**Published:** 2022-04-13

**Authors:** John J. Kopchick, Darlene E. Berryman, Edward O. List

**Affiliations:** 1 Department of Biomedical Sciences, Heritage College of Osteopathic Medicine, Ohio University, Athens OH, 45701, USA; 2 Edison Biotechnology Institute, Ohio University, Athens OH, 45701, USA

**Keywords:** growth hormone receptor, longevity, knockout, growth hormone

One of the most potent interventions used to extend lifespan in laboratory mice is targeted disruption of the growth hormone receptor (GHR). In fact, the current record holder for the Methuselah Mouse Prize for Longevity – a mouse that lived one week shy of five years – is the GHR “knockout” (GHRKO) mouse [1]. A new study by our laboratory suggests that partial knockdown of the GHR beginning at 6 months of age can also extend median and maximal lifespan in female mice [2].

GH secretion decreases with age (referred to as somatopause), causing some to consider the use of GH replacement as a means to counteract aging-related conditions. Counterintuitively, diminished GH action in model organisms, either by way of natural mutations or inactivation of the GH or GHR genes, increases lifespan and slows the aging process through reducing IGF-1, mTOR signaling, and cellular senescence while simultaneously enhancing insulin sensitivity and stress resistance [3]. In addition to lifespan extension, removal of GH action in mice extends healthspan, preventing age-related conditions including frailty, cognitive decline, diabetic nephropathy, diet-induced diabetes, and multiple forms of cancer [3]. While the link between the GH/IGF-1 axis and aging has proven more challenging to demonstrate in humans, evidence continues to grow. For example, individuals with Laron Syndrome (GH insensitivity usually caused by inactivating mutations to the GHR) have reduced mTOR signaling [4], are protected against age-dependent cognitive decline [5], and are resistant to cancer [4,6]. Likewise, some cohorts of isolated GH deficiency (e.g., type 1B in the Brazilian Itabaianinha cohort) have higher numbers of individuals close to and >100 years of age versus the normal population, further suggesting that the findings in rodents are relevant to humans [3].

GHRKO mice (as well as most other mouse lines with reduced GH action) and humans with LS experience the effects of the inactivated GHR gene mutations from conception; thus, the specific impact of GH on longevity in later life required further investigation. Accordingly, our laboratory conducted two separate studies to address this. The first study was published by in 2016 [7] where we suppressed GH action at 1.5 months of age - just prior to sexual development in mice - using an inducible Cre-Lox system to globally disrupt the GHR (termed aGHRKO mice in the original paper but referred to here as 1.5mGHRKO mice). As might be expected with GHR disruption at this younger age, mouse growth is impacted with both body weight and length significantly decreased relative to controls. The most robust disruption of GHR is achieved in liver (>95% decrease) and WAT (~60-90% decrease); while kidney, muscle, and heart demonstrated less efficient disruption. Despite a decrease in body weight, 1.5mGHRKO mice have greater absolute fat mass than controls, and serum IGF-1 and GH levels are significantly decreased and increased, respectively. Importantly, insulin levels are significantly decreased in 1.5mGHRKO mice. Despite only partial disruption of the GHR, female 1.5mGHRKO mice have a significant increase in maximal lifespan. That is, the last female control mouse died at 150 weeks of age while the last 1.5mGHRKD female survived until 177 weeks of age.

Despite these promising results, GHR gene disruption later in life would have greater clinical relevance since interventions initiated at more advanced ages minimize the duration of pharmacological administration without disturbing growth or development. To address this, we conducted a second study recently published [2]. GHR disruption was initiated at 6 months of age – a mature adult age in mice – as opposed to 1.5m in the initial study. Like the first study, female 6mGHRKO mice exhibit a significant extension in lifespan, but this time with mean, median, and maximal lifespan increased compared to controls. Additionally, although 6mGHRKO males did not have a significant increase in lifespan, they did have a median lifespan that nearly reached significance (p=0.07) and had multiple signs of improved healthspan (e.g., decreased cancer, improved insulin signaling, decreased oxidative damage). Importantly, unlike the 1.5mGHRKO mice, both male and female 6mGHRKO mice have no significant changes in bodyweight and minimal impact on body length. Thus, extension in lifespan and healthspan can be achieved with GHR disruption in adult life without major changes in growth.

Collectively, these results suggest that pharmacologic modalities that block GH action later in life, even as somatopause proceeds, could have therapeutic benefit for aging and aging-related diseases. While gene disruption in humans is not viable, approved pharmacological strategies to reduce GH action exist and include somatostatin receptor ligands, dopamine agonists and GH receptor antagonists (GHRAs). Of the options, the one that exclusively targets GH action is the GHRA, pegvisomant. This GHRA, which was discovered in our laboratory with a transgenic mouse line (GHRA mice) and approved by the FDA in 2003, is now used world-wide as a highly effective drug to antagonize GH action in the treatment of patients with acromegaly. Importantly, in a workshop convened to assess development of safe interventions to slow aging and increase healthy lifespan in humans, GHRA is cited as a promising therapeutic [8]. Thus, when considering whether drugs designed to specifically antagonize or inhibit GH action have potential as gerotherapeutics, the current mouse study would suggest “yes”.
